# Rituximab Binding Endows CD20+ Extracellular Vesicles With NK Cell‐Activating Properties in B Cell Lymphoma

**DOI:** 10.1002/jev2.70352

**Published:** 2026-08-09

**Authors:** Adrián V. Otero, Paula Soledad Pérez, Belen Gaete‐Ramírez, Gregorio Cordini, Matías Norte, Enriqueta Martínez, Nicolás Marsol, Cecilia Malusardi, Sofía Rivarola, Ana Cantillo, María Luz Leicaj, Constanza Russo, Juan Quarroz Braghini, Federico Penas, Glenda Ernst, Cecilia Cabral, Silvina Palmer, Manuel Varas‐Godoy, Lourdes Arruvito, Matías Ostrowski

**Affiliations:** ^1^ Instituto de Investigaciones Biomédicas en Retrovirus y SIDA Facultad de Medicina Universidad de Buenos Aires ‐Consejo Nacional de Investigaciones Científicas y Técnicas Ciudad Autónoma de Buenos Aires Argentina; ^2^ Centro de Biología Celular y Biomedicina (CEBICEM), Facultad de Ciencias Universidad San Sebastián Santiago, Santiago Metropolitan Chile; ^3^ División Hematología Hospital de Clínicas “José de San Martín” Ciudad Autónoma de Buenos Aires Argentina; ^4^ Departamento de Cirugía Hospital de Clínicas “José de San Martín” Ciudad Autónoma de Buenos Aires Argentina; ^5^ Servicio de Hematología Hospital Británico Ciudad Autónoma de Buenos Aires Argentina; ^6^ Departamento de Anatomía Patológica Hospital de Clínicas “José de San Martín” Ciudad Autónoma de Buenos Aires Argentina; ^7^ Departamento de Docencia e Investigación Hospital Británico Ciudad Autónoma de Buenos Aires Argentina; ^8^ Centro Ciencia & Vida Fundación Ciencia & Vida Santiago, Santiago Metropolitan Chile

**Keywords:** B cell lymphoma, extracellular vesicles, NK cells

## Abstract

Non‐Hodgkin lymphoma (NHL), predominantly B cell lymphomas (B‐NHL), is currently treated with chemotherapy combined with rituximab (RTX), an anti‐CD20 monoclonal antibody. Despite substantial therapeutic advances, treatment resistance and disease relapse continue to affect a significant fraction of patients. The mechanisms by which the tumour microenvironment and other factors influence RTX efficacy are not fully elucidated. Herein, we hypothesized that CD20+ extracellular vesicles (EVs) shed by B cell lymphomas are recognized by RTX forming immune complexes that modulate natural killer (NK) cell activity via Fcγ receptor (FcγR) interactions. EVs isolated from lymph node explants and plasma samples of B‐NHL patients contained abundant CD20+ vesicles, which were particularly enriched in advanced disease. RTX specifically bound CD20 on these EVs, generating EV‐RTX immune complexes. Functional studies employing EVs from a B‐NHL cell line and from patient‐derived samples demonstrated that, whereas EVs suppressed NK cell activation and cytotoxicity, EV‐RTX immune complexes reversed this inhibitory effect and enhanced NK cell effector functions. Indeed, EV‐RTX immune complexes specifically triggered FcγRIIIa‐dependent NK cell activation, evidenced by increased Syk phosphorylation, CD69 expression, and enhanced lytic activity against target cells. Our findings uncover a previously unrecognized mechanism by which RTX, through the formation of immune complexes with CD20+ EVs, promotes NK cell activation and may enhance therapeutic efficacy. More broadly, these results demonstrate that antibody binding can endow EVs with novel immunomodulatory properties, revealing a potential mechanism by which therapeutic antibodies reshape EV function and influence anti‐tumour immunity.

## Graphical abstract

B cell lymphoma‐derived EVs are intrinsically neutral or suppressive with respect to NK cell activity (panel 1). In the context of rituximab treatment, NK cytotoxicity is enhanced by antibody‐dependent cell‐mediated cytotoxicity (panel 2). Herein we show that, in addition, rituximab recognition of EV‐associated CD20 generates immune complexes that convert them into potent stimulators of NK cells through FcY receptor engagement (panel 3). Thus, therapeutic antibody binding endows tumor‐derived EVs with a previously unrecognized immunostimulatory function that promotes anti‐tumor immunity.

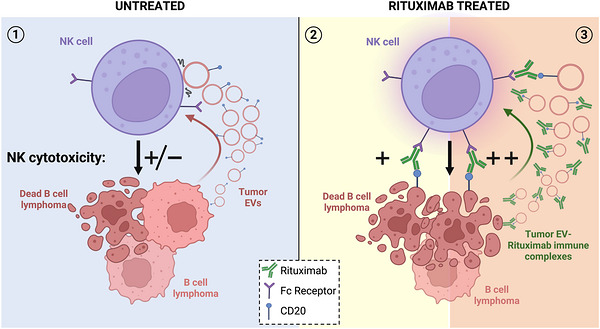

## 1 Background

Non‐Hodgkin lymphoma is a lymphoid tissue tumour, with 85–90% of cases originating from B cells (Silkenstedt et al. 2024). Patients commonly present with progressive lymphadenopathy and symptoms such as fatigue, weight loss, high fever and night sweats. In some cases, extranodal involvement may occur, affecting the bone marrow, central nervous system, or liver. The diagnosis of B‐NHL relies primarily on tissue biopsy (Ansell 2015). Diffuse large B‐cell lymphoma (DLBCL) and follicular lymphoma (FL) together account for nearly half of all B‐NHL cases. DLBCL, the most common subtype in developed countries, is an aggressive disease that requires prompt treatment with curative intent (Alaggio et al. 2022; Silkenstedt et al. 2024). DLBCL and FL are generally chemosensitive, and long‐term survival can be achieved in many patients. In DLBCL, 60%–70% of patients achieve durable remission with the first‐line R‐CHOP regimen (rituximab, cyclophosphamide, doxorubicin, vincristine, and prednisone), whereas the remaining 30%–40% relapse, are refractory to treatment, or discontinue therapy due to toxicity (Crump et al. 2017; Wästerlid et al. 2020). In contrast, FL is typically indolent at diagnosis but follows a relapsing clinical course and, although generally considered incurable, often exhibits prolonged remissions between treatments (Bachy et al. 2019).

CD20 is a B cell specific surface antigen expressed on mature B cells and most B‐NHL but absent from early B cell progenitors and plasma cells (Tedder et al. 1988). Rituximab (RTX), a chimeric monoclonal antibody directed against CD20 which is part of R‐CHOP regimen, has significantly improved clinical outcomes in B‐NHL since its approval in 1997 (Maloney et al. 1997). Its anti‐tumour activity involves several mechanisms, including complement‐dependent cytotoxicity (CDC), antibody‐dependent cell‐mediated cytotoxicity (ADCC) mediated by effector cells such as natural killer (NK) cells and macrophages via Fcγ receptor (FcγR) engagement and the direct induction of apoptosis (Maloney 2012).

Extracellular vesicles (EVs) are nano‐sized, lipid‐bilayered particles released by both normal and tumoural cells, carrying bioactive cargo, including proteins, RNA, and DNA, that closely reflect their parental cells. EVs present in plasma are produced by multiple cell types, including blood cells, and actively released from tissues into the peripheral circulation. Plasma EVs can reach concentrations of >1 × 10^9^ particles/mL (Colombo et al. 2014; Kalluri and LeBleu 2020) and these numbers can be modified during different diseases, such as cancer (Logozzi et al. 2009; Melo et al. 2014, 2015).

Recent studies have highlighted the active role of EVs released by malignant B cells in the pathogenesis of B cell lymphomas. In FL, EVs derived from tumour B cells can reprogram the bone marrow stromal niche, promoting a supportive microenvironment for tumour survival and progression (Dumontet et al. 2021). In DLBCL, tumour‐derived EVs exert a dual effect: while they can enhance dendritic cell vaccine efficacy in vitro, they also promote tumour growth in vivo by modulating the immune response (Chen et al. 2018). Additionally, in Epstein‐Barr virus‐associated lymphomas, EVs function as proinflammatory mediators, contributing to an environment that supports malignant transformation and tumour development (Higuchi et al. 2018). Finally, DLBCL‐derived EVs mediate the intercellular transfer of oncogenic material, including mutated RNAs and oncoproteins (Rutherford et al. 2018). Notably, B cell lymphoma‐derived EVs retain over 70% of the protein content of their parental cells and are enriched in surface markers such as CD19, CD20, and CD22, as well as signalling molecules associated with resistance to chemotherapy and immunotherapy (Yao et al. 2015). Elevated levels of circulating CD20+ EVs may function as decoy antigens by binding RTX, thereby reducing its therapeutic efficacy (Aung et al. 2011). However, the functional consequences associated with the formation of immune complexes between CD20+ EVs and RTX remain completely unexplored.

NK cells play a major role in cancer immune surveillance and are conventionally classified into two main subsets identified by high levels of CD56 and negative for CD16 (CD56^bright^CD16‐) and low levels of CD56 and positive for CD16 (CD56^dim^CD16+). Whereas CD56^bright^CD16‐ NK cells represent a less mature population, CD56^dim^CD16+ NK cells, the major circulating subset in the peripheral blood of healthy individuals, are able to kill tumour cells. The FcγRIIIa (CD16) on NK cells is a critical activating receptor. When NK cells encounter antibody‐coated target cells, FcγRIIIa engagement triggers NK cell activation and degranulation to promote target cell apoptosis by ADCC (Dall'Ozzo et al. 2004).

NK cell activation occurs rapidly after RTX infusion (within 4 h), particularly in individuals with the high‐affinity FcγRIIIa polymorphism and is linked to the mechanism of ADCC (Dall'Ozzo et al. 2004; Veeramani et al. 2011). This activation involves NK cells binding to RTX‐coated target cells, leading to the release of cytotoxic granules and cytokines, along with a decrease in circulating NK cells (Rudnicka et al. 2013). Clinically, the role of NK cells in B‐NHL prognosis is complex. Reduced blood NK cell counts have been associated with poorer patient outcomes in B‐NHL (Klanova et al. 2019), and an expansion of dysfunctional PD‐1‐expressing NK cells (reflecting an exhausted phenotype) has been reported in DLBCL (Vari et al. 2018). Additionally, there is compelling in vivo evidence supporting a key role for NK cell‐mediated ADCC against tumour cells in the lymph nodes of FL patients treated with anti‐CD20 therapies (Wang et al. 2008). However, the prognostic significance of NK cell infiltration in DLBCL lymph nodes remains controversial, as high NK cell densities have been linked to both favourable (Yu et al. 2024) and unfavourable outcomes (Ciavarella et al. 2018). Despite these inconsistencies, NK cell‐engaging therapies like RTX remain a cornerstone of front‐line treatment for B‐NHL patients. In this study, we demonstrate that CD20+ EVs released by B‐NHL cells form immune complexes with RTX, which in turn promotes FcγRIIIa‐dependent activation of NK cells. This phenomenon, consistently observed using a B‐NHL cell line, samples from lymph node explants and plasma from lymphoma patients, reveals a previously unrecognized mechanism through which RTX may enhance anti‐tumour immune responses. More broadly, our findings indicate that antibody binding can fundamentally alter EV function, converting lymphoma‐derived EVs from suppressors of NK‐cell activity into immune‐stimulatory platforms. These observations extend beyond the lymphoma setting and suggest a broader paradigm whereby antibody‐EV interactions shape immune responses, opening new opportunities for the development of EV‐based immunomodulatory strategies.

## 2 Subjects and Methods

### 2.1 Subjects and Samples

This study was conducted in accordance with the Declaration of Helsinki and received approval from the Institutional Review Boards of the Hospital Británico and Hospital de Clínicas “José de San Martín” (Hospital Británico #11973, Facultad de Medicina‐UBA #RESCD‐2023‐3569). Eligible participants included adults aged 18–80 years with histologically confirmed B‐NHL according to the 5th edition of the World Health Organization classification (Alaggio et al. 2022). Lymph node tissue samples were obtained from patients diagnosed with B‐NHL before treatment (*n* = 10) as well as from individuals undergoing surgery for inflammatory, non‐tumoural conditions (non‐B‐NHL, *n* = 11). Both groups were matched for age and sex. The clinical characteristics of the tissue donors are summarized in Table 1.

**TABLE 1:** Characteristics of B‐NHL and non‐B‐NHL tissue donors.

Donor	Age	Gender	Histology	T cells (%)	B cells (%)	Atypical B cells (%)	Plasma cells (%)	Other markers
1	55	Female	FLH	38.6	55.4	no	3.5	
2	64	Male	FLH	86.0	3.6	no	0.6	
3	65	Male	AC	53.9	36.6	no	1.6	
4	24	Male	FLH	72.1	20.0	no	0.3	
5	52	Female	FLH	54.6	41.0	no	0.8	
6	71	Male	DLBCL	65.9	32.5	31.8	0.1	CD19+, CD20++, CD10+, CD38+, CD5−, CD79b+
7	27	Female	FL	14.8	84.7	82.72	0.1	CD19+, CD20++, CD10+, CD81+, CD38+, CD5−
8	79	Female	FL	58.1	40.9	40.6	0.0	CD19−/+, CD20++, CD5−, CD10+, CD79b+, CD81+
9	77	Male	FL	15.8	83.9	83.9	0.1	CD19−/+, CD20++, CD38−, CD10+, CD79b+, CD200+
10	78	Female	DLBCL	2.4	91.9	91.9	0.0	CD19+, CD20++, CD38−/+, CD10−/+, CD79b+, CD200−/+
11	35	Male	DLBCL	28.9	65.9	64.0	0.0	CD19+, CD20++, CD10+, CD38+, CD5−, CD79b+
12	52	Female	FLH	NA	NA	NA	NA	
13	63	Male	LT	NA	NA	NA	NA	
14	47	Female	GI	NA	NA	NA	NA	
15	72	Male	HL	59.1	25.0	no	3.7	
16	22	Female	HF	59.9	37.8	no	0.1	
17	37	Female	FLH	24.4	39.1	no	0.1	
18	71	Female	MCL	7.2	91.95	91.7	0.0	CD19++, CD20+++, CD81++, CD10−, CD5++, CD11c−
19	71	Female	DLBCL	9.4	89.3	87.9	0.0	CD19++, CD20+++, CD10++, CD81+, CD5−, CD38++
20	44	Female	FL	NA	NA	NA	NA	
21	62	Male	FL	NA	NA	NA	NA	

*Note*: All donors were untreated at the time of sampling. Samples #1 to #11 were used for correlation analysis, while the remaining samples were used for functional experiments. Abbreviations: AC metastasis, Adenocarcinoma metastasis; DLBCL, Diffuse large B cell lymphoma; FL, Follicular lymphoma; FLH, Follicular lymphoid hyperplasia; GI, Granulomatous Inflammation; MCL, Mantle cell lymphoma; NA, not available.

In addition, plasma samples were collected from patients who were recently diagnosed with or relapsed B‐NHL (*n* = 15). These samples were collected immediately before starting treatment. Healthy donor plasma samples (HD, *n* = 20) were obtained from the Blood Bank. Both patient cohorts were matched for age and sex. The clinical data for the plasma donors are presented in Table 2.

**TABLE 2:** Characteristics of B‐NHL plasma donors.

Patient	Age	Gender	Histology	Stage	IPI	Status at sampling	Treatment response	Regimen
1	68	Male	DLBCL	II	3	untreated	PD	R‐CHOP
2	49	Female	FL	I	1	untreated	CR	R‐CEOP
3	42	Male	FL	IIA	1	untreated	CR	R‐CHOP
4	53	Female	FL	IVA	1	untreated	CR	R‐B
5	68	Male	MCL	IVA	2	untreated	CR	R‐B
6	69	Male	FL	IVA	2	relapse	CR	R‐B
7	70	Male	FL	IA	2	relapse	CR	R‐B
8	67	Female	FL	IVA	2	relapse	PD	R‐B
9	49	Female	FL	IIIB	2	untreated	CR	R‐CHOP
10	65	Female	DLBCL	IIIA	3	untreated	CR	R‐CHOP
11	46	Female	DLBCL	IVA	3	untreated	CR	R‐CHOP
12	63	Male	MCL	IVA	3	untreated	PD	R‐CHOP
13	48	Male	DLBCL	IIA	3	untreated	PD	R‐CHOP
14	61	Male	DLBCL	IVA	3	untreated	Death	R‐CHOP
15	74	Female	FL	IIIA	3	relapse	CR	R

*Note*: All patients had active diseases, including Diffuse large Bcell lymphoma (DLBCL), Follicular lymphoma (FL), or Mantle cell lymphoma (MCL), classified according to the Ann Arbor staging system and the international prognostic index (IPI) at the time of blood sampling. Eight cases were treatment‐naïve (untreated), and three cases had relapsed prior to initiation of salvage therapy (relapse). Treatment responses were categorized as complete response (CR), partial response (PR) and progressive disease (PD). The regimens used included R‐CHOP (rituximab, cyclophosphamide, doxorubicin, vincristine, prednisolone), R‐CEOP (rituximab, cyclophosphamide, etoposide, vincristine, prednisolone), R‐B (rituximab, bendamustine) and R (rituximab).

### 2.2 Lymph Node Tissue Culture

Tissue was processed immediately after resection. A portion of fresh tissue was used for tumour immunophenotyping by flow cytometry. Then, small pieces (250 mg) of lymph node tissue (explants) were prepared using a scalpel. Explants were placed in a 48‐multiwell plate (GBO, #677102) containing 0.5 mL serum‐free RPMI 1640 (GIBCO, #21870076) medium and incubated at 37°C. After 24 h, supernatants and cells were collected for EVs purification and flow cytometry, respectively.

### 2.3 Plasma Samples Processing

Approximately 10 mL of whole blood samples were obtained from healthy and B‐NHL fasted donors using EDTA‐containing vacutainer tubes (BD Biosciences, #367863). After being centrifuged for 10 min at 300 × *g*, plasma was separated and then depleted from platelets by centrifugation at 2000 × g for 15 min with addition of 200 nM prostaglandin I2 (PGI2, Cayman Chemical, #18220) to inhibit platelet activation. Finally, aliquots were stored at −80°C until use.

### 2.4 Cell Line Culture

Ramos B cell line (ATCC, RRID:CVCL_1646) and K562 cell line (ATCC, RRID:CVCL_K562) were cultured in RPMI 1640 (GIBCO, #21870076) supplemented with 10% heat‐inactivated foetal calf serum (FCS, Sigma–Aldrich, #G7513), 2 mM L‐Glutamine (Sigma‐Aldrich), penicillin and streptomycin (Sigma–Aldrich, #P0781). Cells were incubated in 95% air and 5% CO2 at 37°C. For cell line‐derived EVs isolation, 3.5 × 10^8^ cells at final concentration of 5 × 10^6^ cells/mL (trypan blue determined viability >95%) were washed twice and seeded in RPMI 1640 supplemented with 10% EV‐depleted FCS for 2 days. Afterward, cell supernatant was harvested and EVs were purified. To prepare RPMI 1640 depleted of EVs, RPMI 1640, supplemented with 2 mM L‐Glutamine, penicillin, streptomycin and 30% heat‐inactivated FCS, was ultracentrifuged at 100,000 *g* overnight at 4°C (Sorvall WX 100+, angle rotor T‐865, k‐factor = 51.7, Themo Scientific). The supernatant was filtered through a 0.22 um filter and diluted with serum‐free RPMI 1640 to reach the final serum concentration of 10%.

### 2.5 NK Cells Isolation

Primary NK cells were isolated from buffy coats obtained from 1 unit of blood from volunteers’ donations provided by the Blood Bank at Hospital de Clinicas “Jose de San Martín”, Buenos Aires. NK cells were purified by negative selection using RosetteSep NK cell enrichment kit (STEMCELL Technologies, #15065) following the manufacturer instructions. Cells were incubated overnight at 37°C with 5% CO2 in RPMI 1640 supplemented with 10% heat‐inactivated FCS, glutamine and antibiotics prior to assay usage. Purity of isolated cells was always above 90%, as assessed by flow cytometry (CD3−CD56+ cells).

### 2.6 EVs Isolation

Tissue‐derived extracellular vesicles (tEVs) were purified from 250 mg of tissue, with each sample resuspended in a final volume of 0.5 mL. Duplicates or triplicates were prepared depending on the size of the original tissue. Separately, Ramos‐derived EVs were isolated from 3.5 × 10^8^ cells in a final volume of 70 mL. Both types of EVs were then purified from cell culture supernatants by sequential centrifugation. Cell contamination was removed from culture supernatant by centrifugation at 300 × *g* for 10 min. To remove apoptotic bodies and large cell debris, the supernatants were then spun at 2000 × *g* for 10 min, followed by centrifugation at 10,000 × *g* for 30 min to remove large vesicles (Sorvall WX 100+, Thermo Scientific, angle rotor T‐865, k‐factor = 51.7, for Ramos supernatant; Centrifuge 5424 R, Eppendorf for tissue supernatant). Finally, small EVs were collected by ultracentrifugation at 100,000 × *g* for 90 min twice (Sorvall WX 100+, Thermo Scientific, angle rotor T‐865, k‐factor = 51.7, for Ramos supernatant; angle rotor TFT 80.2, k‐factor = 22.6, for tissue supernatant). EVs were resuspended in phosphate buffer saline (PBS) and stored at −80°C until used. Plasma EVs (pEVs) were purified by size exclusion chromatography (SEC) as previously described (Adamczyk et al. 2023). Briefly, Sepharose CL‐2B columns (Cytiva, #17014001) were assembled using 12‐mL empty cartridges with 20‐mm hydrophobic frits (Applied Separations, #2419). Two mL of plasma were loaded on top, and 1 mL fractions were eluted using 0.9% NaCl–0.38% sodium citrate solution. Fractions containing pEVs (fractions 4‐5‐6) were centrifuged at 30,000 × *g* at 4°C for 90 min in a centrifuge (Biofuge Stratos, angle rotor Heraeus, k‐factor = 340). Centrifugation of pEV‐enriched SEC fractions at 30,000 *g* for 90 min was validated by WB to be equivalent to centrifugation at 100,000 g (Figure S1) (Adamczyk et al. 2023). Pellets were pooled, resuspended in 60 µL of PBS and stored at −80°C until used.

### 2.7 Nanoparticle‐Tracking Analysis (NTA)

Size distribution and concentration of purified EVs were analyzed using the ZetaView Twin equipment (Particle Metrix) at Instituto de Investigaciones en Microbiología y Parasitología Médica (IMPaM), Universidad de Buenos Aires‐CONICET. All samples were diluted in PBS to a final volume of 1 mL (dilution 1:1000). For each measurement, two cycles were performed under the following settings: Focus: autofocus; Camera sensitivity for all samples: 80; Shutter: 100; Cell temperature: 25°C; Laser wavelength: 488 nm; Filter wavelength: scatter. After capture, videos were analysed by ZetaView Software 8.05.16 with specific analysis parameters: Maximum particle size: 1000, Minimum particle size: 10, Minimum particle brightness: 30.

### 2.8 Immunohistochemistry

Lymph node tissue was fixed in 10% formaldehyde, paraffin‐embedded and sectioned by the Pathology Department, Hospital de Clínicas “José de San Martín”. Tissue sections (4 µm) were mounted on adhesive glass slides, deparaffinized in xylene (2 × 5 min) and rehydrated in graded ethanol. Endogenous peroxidase was quenched, and epitope retrieval was performed. Non‐specific binding was blocked with 5% bovine serum albumin (BSA). CD20+ cells were detected using a primary anti‐CD20 antibody (dilution 1:500, clone L26, Cell Marque, #120M‐8) for 1 h at 4°C, followed by PBS washes. Immunostaining and haematoxylin counterstaining were performed using the Ventana BenchMark XT (Roche), a fully automated system with an HRP multimer‐based DAB detection kit (UltraVIEW, Ventana). Slides were examined under a Nikon Eclipse E200 microscope at 4× and 400× magnification.

### 2.9 Western Blotting

For characterization, Ramos‐derived EVs, tEVs and pEVs were isolated according to the above sections. Protein samples were quantified with Pierce BCA Protein Assay Kit (Thermo Fisher, #23225). Twenty µg of tEVs and Ramos‐derived EVs as well as 20 µL of pEVs were lysed in Laemmli buffer and later loaded. Cell lysates of tissue and Ramos cells (20 µg) as well as previously platelet depleted plasma (1 µL) were used as control of purity. Equal volumes of extracts were separated on 12% SDS‐PAGE under non‐reducing conditions for CD20 (R&D systems, #MAB42252), CD63 (BD Pharmingen, #556019), CD81 (BD Pharmingen, #555675) and IgG (Jackson Immunoresearch, #109‐035‐003) detection, 10% SDS‐PAGE under reducing conditions for Syntenin‐1 (Genetex, #GTX108470), GRP94 (Enzo Life Sciences, #ADI‐SPA‐850), ALIX (Cell Signalling Technology, #92880), HSP70 (Enzo Life Sciences, #ADI‐SPA‐810), and APOA1 (Cell Signalling Technology, #3350S), and further blotted on PVDF transfer membrane. Primary antibody dilution used for blotting was 1:10000 for IgG and 1:1000 for the rest of the antibodies. Anti‐species secondary antibodies conjugated to HRP were used at 1:10000 dilution. All blots were revealed using SuperSignal West Pico PLUS Chemiluminescent Substrate (Thermo Fisher, #34577), and images were acquired with the BioSpectrum‐815 Imaging System (UVP).

### 2.10 Transmission Electron Microscopy (TEM)

The purified EVs suspension was fixed in 2% paraformaldehyde and subjected to a negative staining protocol. To obtain TEM images, 10 µL of EVs suspensions were dropped on Formvar‐coated nickel grids for 20 min. After that, EVs adsorbed on the grids were washed with PBS (pH 7.4) and postfixed in 1% glutaraldehyde. The grids were rinsed with dH2O and then contrasted successively with 2% uranyl acetate (pH 7), and 2% methylcellulose/0.4% uranyl acetate (pH 4) on ice. EVs were visualized using a transmission electron microscope Hitachi HT 7800 (Hitachi, Tokyo, Japan) operated at 80 kV and photographed with a NS 15 AMT camera (Advanced Microscopy Techniques Corp., Woburn, MA, USA) at the Transmission Electron Microscopy Facility of Instituto de Investigaciones en Ciencias de la Salud (INICSA)‐CONICET, Facultad de Ciencias Médicas, Universidad Nacional de Córdoba. In order to determine RTX binding, EVs were stained with RTX (1 µg/mL, Mabthera). RTX binding was detected by immunogold labelling with protein A conjugated to 10 nm colloidal gold particles. Briefly, EVs preparation on Formvar‐carbon coated EM grids, were transferred to a drop of 1% BSA blocking buffer and then incubated with protein A‐gold (dilution 1:15) for 60 min. After successive washes the grids were contrasted and visualized as explained above. The number of gold particles associated with EVs were counted for all the structures compatible with EVs observed in two grids over more than 100 EVs and expressed as mean ± standard error of mean (SEM).

### 2.11 Flow Cytometry

To detect EVs by flow cytometry, anti‐human CD63 antibody (BD Biosciences, #556019) was coupled to 4 µm aldehyde‐sulphate beads (Invitrogen, #A37306) by incubating 25 µg of antibody with 1 × 10^8^ beads followed by blocking of remaining activated groups with PBS‐4% BSA. Cell culture supernatants were cleared by centrifugations at 1200 rpm (for 5 min) and 4,000 rpm (for 20 min). Cleared supernatants (100 µL) and/or plasma EVs (dilution 1:50) were incubated with 20,000 (0.1 µL) anti‐CD63 coupled beads overnight at 4°C with 700 rpm shaking. Beads were washed twice in PBS‐2% BSA and incubated with PECy7 anti‐CD81 (#349512), FITC anti‐CD107a (#328606) and Alexa Fluor 647 anti‐CD20 (#302318; all from Biolegend) for 30 min on ice. The binding of RTX (1 µg/mL, Mabthera) in both immunoadsorbed EVs and cell lines was revealed by using Alexa Fluor 647 anti‐human IgG Fc (Biolegend, #410714). Beads were acquired using a Northern Lights (Cytek) flow cytometer and analysed with FlowJo 10.6.2. Threshold of negative staining was obtained with beads incubated with unconditioned EV‐depleted medium.

Tumour immunophenotyping was performed at Flow Cytometry Core, Haematology Department at Hospital de Clínicas “José de San Martín” according to EuroFlow standard operating procedures.

### 2.12 Phosphorylation of Syk Assay

To analyse Syk phosphorylation, purified NK cells were exposed to Ramos cell‐derived EVs alone (5 µL of purified EVs representing ∼4.1 × 10^8^ EVs), immune complexes (EVs plus RTX, IC) and/or RTX alone (1 µg/mL) or non‐exposed for 5 min at 37°C. Immune complexes were made by mixing 5 µL of EVs with RTX (1 µg/mL) for 1 h at 37°C in PBS. To eliminate RTX unbound to EVs, the mixture was filtered by using Vivaspin 500 centrifugal concentrators 300 kDa MWCO (Sartorius, #VS0151). Stimulation with anti‐CD16 mAb 10 µg/mL (STEMCELL Technologies, clone 3G8, #60041) exposed for 15 min at 4°C, then plus goat anti‐mouse IgG F(ab’)_2_ 50 µg/mL for 10 min at 4°C (Jackson Immunoresearch, #115‐006‐068) was used as positive control. Cells were then stained with a mAb directed to phosphorylated Zap70 (Y319)/Syk (Y352) after cell fixation and permeabilization (BD Biosciences, #557817). Data were analysed by flow cytometry.

### 2.13 NK Cell Activation

Briefly, purified NK cells (1 × 10^5^) were stimulated with suboptimal doses of IL‐15 (5 ng/mL, Peprotech, #200‐15) and exposed to Ramos cell‐derived EVs alone (5 µL of purified EVs representing ∼4.1 × 10^8^ EVs), immune complexes (EVs plus RTX) and/or RTX alone (1 µg/mL) or non‐exposed for 4 h at 37°C. Following incubation, cells were surface stained with anti‐CD56 Brilliant Violet 506 (#318334), anti‐CD3 APC‐Cy7 (#300318) and anti‐CD69 Alexa Fluor 647 (#310918) all from Biolegend. NK cells were defined as CD3‐CD56+ cells. The frequency of CD69+ activated NK cells was analysed by flow cytometry. When indicated, NK cell activation was also assessed in the presence of tEVs (∼8.9 × 10^9^) and/or pEVs (∼1.1 × 10^9^).

### 2.14 NK Cell‐Mediated Cytotoxicity

A 4‐h standard cytotoxicity assay was performed by using CFSE‐labelled human erythroleukemic cell line (K562) as target cells, mixed with NK cells as effector cells. Briefly, purified NK cells (2.5 × 10^5^ cells) were exposed to Ramos cell‐derived EVs alone (5 µL of purified EVs representing ∼4.1 × 10^8^ EVs), immune complexes (EVs plus RTX), RTX (1 µg/mL) or non‐exposed for 30 min at 37°C. Thereafter, CFSE labelled K562 cells (5 × 10^4^) (Invitrogen, #C34554) were added at 5:1 Effector:Target ratio. After 4 h, cells were harvested, labelled with Zombie NIR (Biolegend, #423105), and analysed by flow cytometry. In some experiments NK cells were previously exposed to Fc‐Blocking (Biolegend, #422302). Target cells alone and/or cells exposed to EVs were used as controls. When cytotoxic effect on Ramos cells was evaluated, purified NK cells were exposed to ∼4.1 × 10^8^ Ramos‐derived EVs, IC (∼4.1 × 10^8^ EVs + RTX), RTX (1 µg/mL), IC plus free‐RTX or non‐exposed for 30 min at 37°C. As non‐specific antibody control, purified NK cells were exposed to ∼4.1 × 10^8^ Ramos‐derived EVs, EVs pre‐incubated with Trastuzumab (∼4.1 × 10^8^ EVs + TZ 1 µg/mL, Herceptin), TZ (1 µg/mL), EVs+TZ plus free‐TZ or non‐exposed for 30 min at 37°C. Thereafter, CFSE‐labeled Ramos cells were added at 5:1 NK: Ramos ratio. After 4 h, cells were labelled with Zombie NIR and analysed by flow cytometry. Ramos cells alone and/or exposed to EVs and RTX or TZ were used as controls. Percentage of cytotoxicity was calculated as 100 × percentage of CFSE+Zombie NIR high cells / percentage of CFSE+ cells.

### 2.15 Statistics

Statistical analyses were performed using GraphPad Prism 8.0 software. Data normality was evaluated by Shapiro–Wilk test. For comparisons between groups, Wilcoxon signed rank test, Kruskal–Wallis test, Friedman test, or one‐way ANOVA test was used, as appropriate. Correlation was assessed using Spearman correlation test. A *p* value <0.05 was considered statistically significant.

## 3 Results

### 3.1 Recognition of Lymphoma Cell‐Derived CD20+ EVs by Rituximab

**FIGURE 1:**
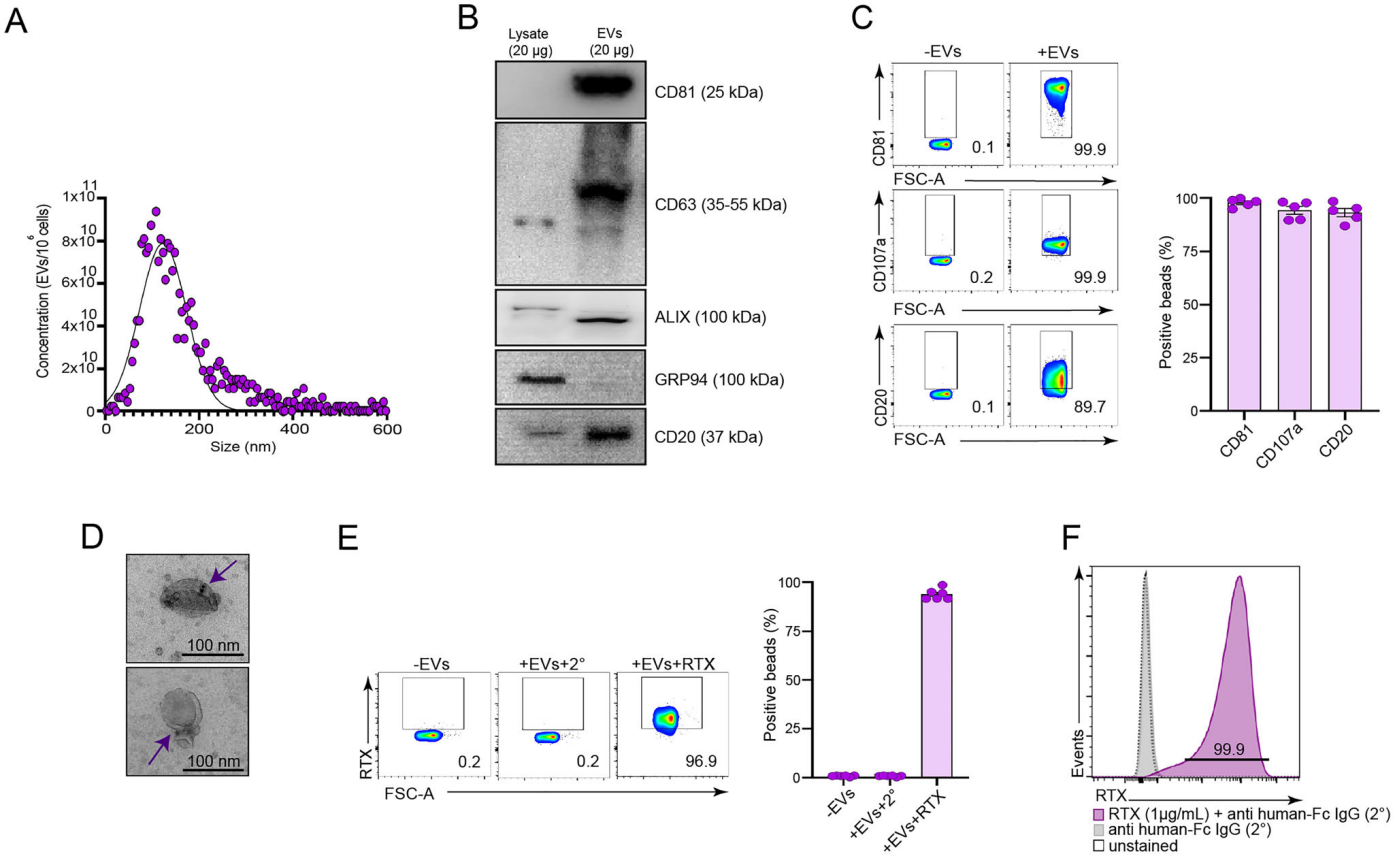
**CD20+ EVs derived from B cell lymphoma cells bind RTX creating immune complexes. (A)** Representative curve of size distribution of EVs from B cell lymphoma Ramos cell line (*n* = 4) obtained by NTA. **(B)** Representative immunoblot characterization of EVs derived from Ramos cells (20 µg) using antibodies directed against the EV markers CD81, CD63 and ALIX, non‐EV marker GRP94 and CD20. Cell lysates were used as control (20 µg). One is shown out of three performed. **(C)** Flow cytometry of Ramos‐derived EVs captured on anti‐CD63‐coated beads (CD81, CD107a and CD20). Representative dot plots (left) and bars showing the percentage of positive beads for each marker (right) are shown (*n* = 5). Latex beads exposed to unconditioned EV‐depleted media were used as negative control. **(D)** Representative TEM of EVs from Ramos showing protein A‐immunogold staining to detect the binding of rituximab (scale 100 nm). **(E)** Binding of RTX (1 µg/mL) to Ramos‐derived EVs using bead‐based flow cytometry. Representative dot plots (left) and bars showing the percentage of positive beads for RTX (right; *n* = 6) are shown. Latex beads exposed to unconditioned EV‐depleted media were used as negative control. **(F)** Binding of RTX (1 µg/mL) to Ramos cells analysed by flow cytometry. Representative histograms from three experiments are shown. Mean ± SEM of n experiments are shown in **C, E** (right). Replicates correspond to different EV preparations.

To evaluate the hypothesis that EV‐RTX immune complexes modulate NK cell activity during B cell lymphoma, we first tested whether RTX could recognize lymphoma cell‐derived EVs. EVs were purified from the human B cell lymphoma line Ramos, using a classical protocol of differential centrifugation (Théry et al. 2006). EVs characterization was performed according to the MISEV 2023 guidelines (Welsh et al. 2024). Nanoparticle tracking analysis (NTA) revealed a size distribution compatible with small extracellular vesicles (Figure 1A). The mean ± standard error of mean (SEM) diameter of recovered EVs from Ramos cells was 132.4 ± 16 nm. In addition, particle concentration per 10^6^ cells was ∼1.6 ± 0.4 × 10^10^. Immunoblot showed that EVs preparations were enriched in the canonical EV markers CD81 and CD63 (tetraspanins, transmembrane proteins) as well as ALIX (cytosolic), compared to cell lysates. In contrast, the endoplasmic‐reticulum‐resident protein GRP94 (endoplasmin) was not detected in the EVs fractions (Figure 1B), indicating minimal contamination by intracellular components and supporting the quality of the EV preparations. Since CD20 is the target of RTX, we next evaluated its presence in Ramos‐derived EVs. Western blot analysis showed that CD20 was detected in cell lysates and was enriched in EV fractions, demonstrating that secreted EVs retain the target antigen recognized by RTX (Figure 1B). To further confirm the surface exposure of CD20 on Ramos‐derived EVs, vesicles isolated from culture supernatants were captured onto anti‐CD63‐coated beads and analysed by flow cytometry following staining with a fluorescent anti‐CD20 antibody. Ramos‐derived EVs were readily recognized by the anti‐CD20 antibody, indicating that CD20 epitopes are exposed and accessible on the EV surface (Figure 1C). The EV‐associated proteins CD81 and CD107a were used as positive controls. Quantitative analysis revealed that 93.2 ± 1.9% of CD63‐captured EVs were positive for CD20, demonstrating that this therapeutic target is highly represented on the surface of Ramos‐derived EVs (Figure 1C). Importantly, this also demonstrates that the CD20 molecules present on Ramos‐derived EVs retain surface‐exposed epitopes recognized by the therapeutic antibody rituximab. RTX binding to EVs was confirmed by immunogold‐TEM (Figure 1D) and bead‐based flow cytometry analyses (Figure 1E), indicating that EV‐associated CD20 is accessible for antibody recognition. As expected, RTX also recognized CD20 present on the cell surface (Figure 1F). Altogether, these results indicate that RTX not only recognizes cellular CD20, but it also recognizes CD20+ EVs produced by lymphoma cells.

### 3.2 CD20+ EV‐RTX Immune Complexes Potentiate NK Cell Activation and Cytotoxicity

We then investigated whether RTX‐specific recognition of CD20 on the surface of lymphoma‐derived EVs modified the immunomodulatory properties of the EVs on NK cell activation and cytotoxicity.

**FIGURE 2:**
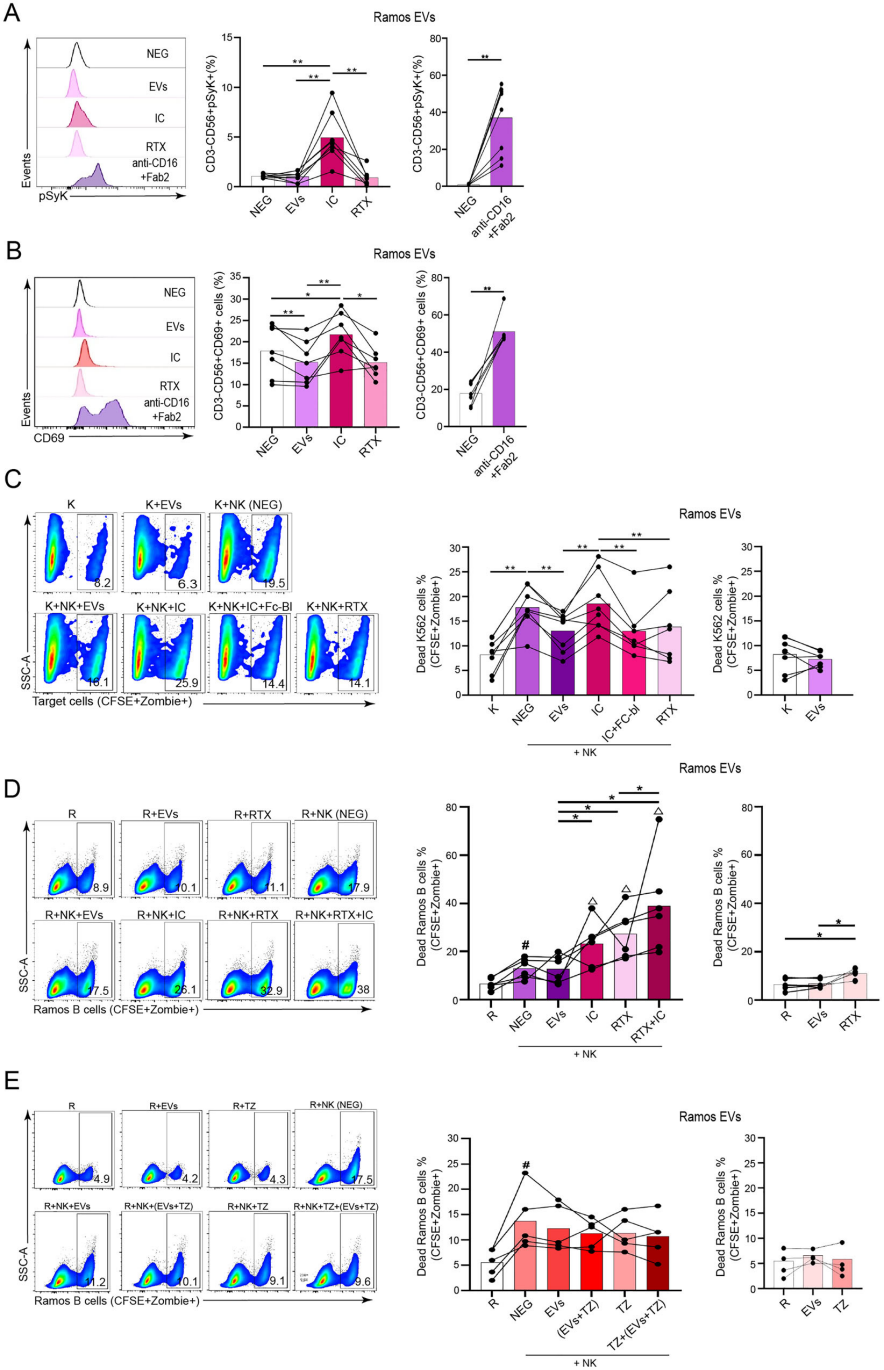
**CD20+ EV‐RTX immune complexes potentiate NK cell activation and cytotoxicity. (A)** Purified NK cells from healthy donors (HD) were exposed to Ramos‐derived EVs, immune complexes (IC) (∼4.1 × 10^8^ EVs + RTX), RTX (1 µg/mL) or unexposed (NEG) for 30 min and the frequency of CD3‐CD56+pSyk+ NK cells was analysed by flow cytometry. Representative histograms (left) and the percentage of positive cells (middle) are shown (*n *= 8). Stimulation with anti‐CD16 mAb 10 µg/mL for 15 min at 4°C plus goat anti‐mouse IgG F(ab’)_2_ 50 µg/mL for 10 min at 4°C was used as positive control (right). **(B)** Purified NK cells from HD were stimulated with suboptimal doses of IL‐15 (5 ng/mL) and then exposed to (∼4.1 × 10^8^ Ramos‐derived EVs, IC (∼4.1 × 10^8^ EVs + RTX), RTX (1 µg/mL) or non‐exposed (NEG) for 4 h. The frequency of CD3‐CD56+CD69+ NK cells were analysed by flow cytometry. Representative histograms (left) and the percentage of positive cells (middle) are shown (*n* = 7). Stimulation with anti‐CD16 mAb 10 µg/mL for 15 min at 4°C plus goat anti‐mouse IgG F(ab’)_2_ 50 µg/mL for 10 min at 4°C was used as positive control (right). **(C–E)** Purified NK cells were incubated with the indicated treatments for 30 min at 37°C. Subsequently, the indicated CFSE‐labelled target cells were added at a 5:1 effector‐to‐target ratio and co‐cultured for 4 h. Cells were then stained with Zombie NIR and analysed by flow cytometry. Target cells without NK exposed to the indicated treatments were used as controls (right). When indicated, Fc‐blocking was included as an additional control. Cytotoxicity was calculated as 100 × (% CFSE+ Zombie NIR high cells/% CFSE+ cells). Representative dot plots (left) are shown together with the quantification of target cell death (middle). **(C)** NK cells (*n* = 7) were exposed to ∼4.1 × 10^8^ Ramos‐derived EVs, IC (∼4.1 × 10^8^ EVs + RTX), RTX (1 µg/mL), or non‐exposed (NEG). CFSE‐labelled K562 cells (indicated with a K) were used as targets. **(D)** NK cells (*n* = 6) were exposed to ∼4.1 × 10^8^ Ramos‐derived EVs, IC (∼4.1 × 10^8^ EVs + RTX), RTX (1 µg/mL), IC plus free RTX, or non‐exposed (NEG). CFSE‐labelled Ramos B cells (indicated with an R) were used as targets. **(E)** NK cells (*n* = 5) were exposed to ∼4.1 × 10^8^ Ramos‐derived EVs, ∼4.1 × 10^8^ EVs pre‐incubated with trastuzumab (EVs+TZ), TZ (1 µg/mL), EVs + TZ plus free TZ, or non‐exposed (NEG). CFSE‐labelled Ramos B cells (indicated with an R) were used as targets. *p* values were determined by Friedman and Wilcoxon matched‐pairs signed rank test. *p < 0.05 and **p < 0.01. # *p* < 0.05 regarding Ramos cells without NK (R). Δ *p* < 0.05 regarding unmodulated cytotoxicity (NEG). Replicates correspond to different NK donors.

We first evaluated early FcγR signalling by measuring phosphorylation of Syk in purified NK cells from healthy donors (HD). The following experimental stimuli were assessed: (i) Ramos‐derived EVs, (ii) RTX alone, (iii) Ramos‐derived EVs coated with RTX, (iv) a therapeutic anti‐CD16 mAb (positive control). As expected, RTX alone or EVs alone had negligible effects on pSyk levels (0.9 ± 0.18% and 0.9 ± 0.3%, respectively). Remarkably, EV‐RTX immune complexes (IC) significantly enhanced Syk phosphorylation (5.0 ± 0.8%, *p* < 0.01; Figure 2A, histograms in the left panel, quantification in the middle panel). CD16 crosslinking was used as a positive control (Figure 2A, right panel).

To evaluate the activation status of NK cells, we quantified the cell‐surface expression of the activation marker CD69 (Figure 2B). NK cells were stimulated with suboptimal doses of IL‐15 and then exposed to different experimental conditions. Ramos‐derived EVs reduced CD69 expression compared with unstimulated controls, indicating a potential inhibitory effect on NK cell activation (15.2 ± 1.9% vs. 17.9 ± 2.2%, *p* < 0.01; Figure 2B left and middle panels). Importantly, this EV‐mediated inhibitory effect was reversed when EVs formed an immune complex with RTX (IC), showing a significant increase in CD69 expression (21.7 ± 1.9%, *p* < 0.01). As expected, addition of RTX alone did not modify CD69 expression, whereas engagement of CD16 triggered a marked increase in CD69 surface levels (Figure 2B, right panel), confirming the responsiveness of NK cells under these experimental conditions.

Next, we evaluated the impact of lymphoma‐derived EVs and EV‐RTX immune complexes on the cytotoxic activity of purified NK cells. K562 cells, an immortalized human lymphoblast cell line derived from a patient with chronic myelogenous leukaemia, were used as targets because their lack of MHC class I expression renders them highly sensitive to NK cell‐mediated cytotoxicity (West et al. 1977). The addition of Ramos‐derived EVs to K562 cells did not modify cell viability (Figure 2C, right panel). However, the addition of these EVs in the presence of NK cells significantly reduced NK cell cytotoxicity (13.4 ± 1.5%) compared with baseline levels (18.0 ± 1.6%, *p* < 0.01; Figure 2C, left and middle panels). Remarkably, this suppressive effect was completely reversed by EV‐RTX immune complexes, which restored NK cell cytotoxicity to baseline levels (18.9 ± 2.3%). Importantly, blockade of Fcγ receptors completely abrogated the ability of EV‐RTX immune complexes to restore NK cell activity (13 ± 2%, *p* < 0.01), demonstrating that this effect is mediated through FcγR signalling. Since K562 cells do not express CD20 (Schneider, 2017), addition of RTX alone had no effect on NK cell cytotoxic activity (13.6 ± 2.0%, *p* < 0.01) (Figure 2C, left and middle panels).

In our final experimental setting, cytotoxicity was directly assessed on Ramos cells (tumour B cells) (Figure 2D, left and middle panels). Under these conditions, Ramos‐derived EVs did not impair NK cell functionality. However, when these EVs were complexed with RTX, a significant increase in NK‐mediated cytotoxic activity was observed (23.2 ± 3.8%, *p* < 0.05). Although no statistically significant differences were detected when comparing the effect of EV‐RTX immune complexes with RTX alone (1 µg/mL), this effect is likely explained by the direct action of RTX on CD20‐expressing Ramos cells (Figure 2D, right panel). Notably, the simultaneous addition of RTX and EV‐RTX immune complexes resulted in an additive effect, leading to a marked increase in tumour cell death (39.0 ± 8.0%, *p* < 0.05), which exceeded the cytotoxicity observed with either condition alone (Figure 2D, left and middle panels).

Finally, to confirm that NK cell activation depends on the specific recognition of EV‐associated CD20 by RTX and the consequent formation of an EV‐bound immune complex, rather than the mere presence of an IgG antibody on the EV surface, we repeated the cytotoxicity assay using EVs pre‐incubated with trastuzumab. Trastuzumab is a humanized IgG1 monoclonal antibody that specifically targets HER2/ErbB2 and is widely used for the treatment of HER2‐positive breast and gastric cancers. Since Ramos cells used as EV source do not express HER2 (Figure S2), any interaction between trastuzumab and Ramos‐derived EVs would be expected to be nonspecific. Consistent with this, neither trastuzumab‐incubated EVs, trastuzumab alone, nor the combination of both enhanced NK cell cytotoxicity against Ramos B cells as target (Figure 2E, left and middle panels).

Taken together, our results indicate that the specific recognition of lymphoma‐derived EVs by RTX leads to the formation of immune complexes that engage Fcγ receptors on NK cells, thereby enhancing their activation and tumour cell killing.

### 3.3 CD20+ EVs From B‐NHL Lymph Node Explants Bind RTX and Activate NK Cells

**FIGURE 3:**
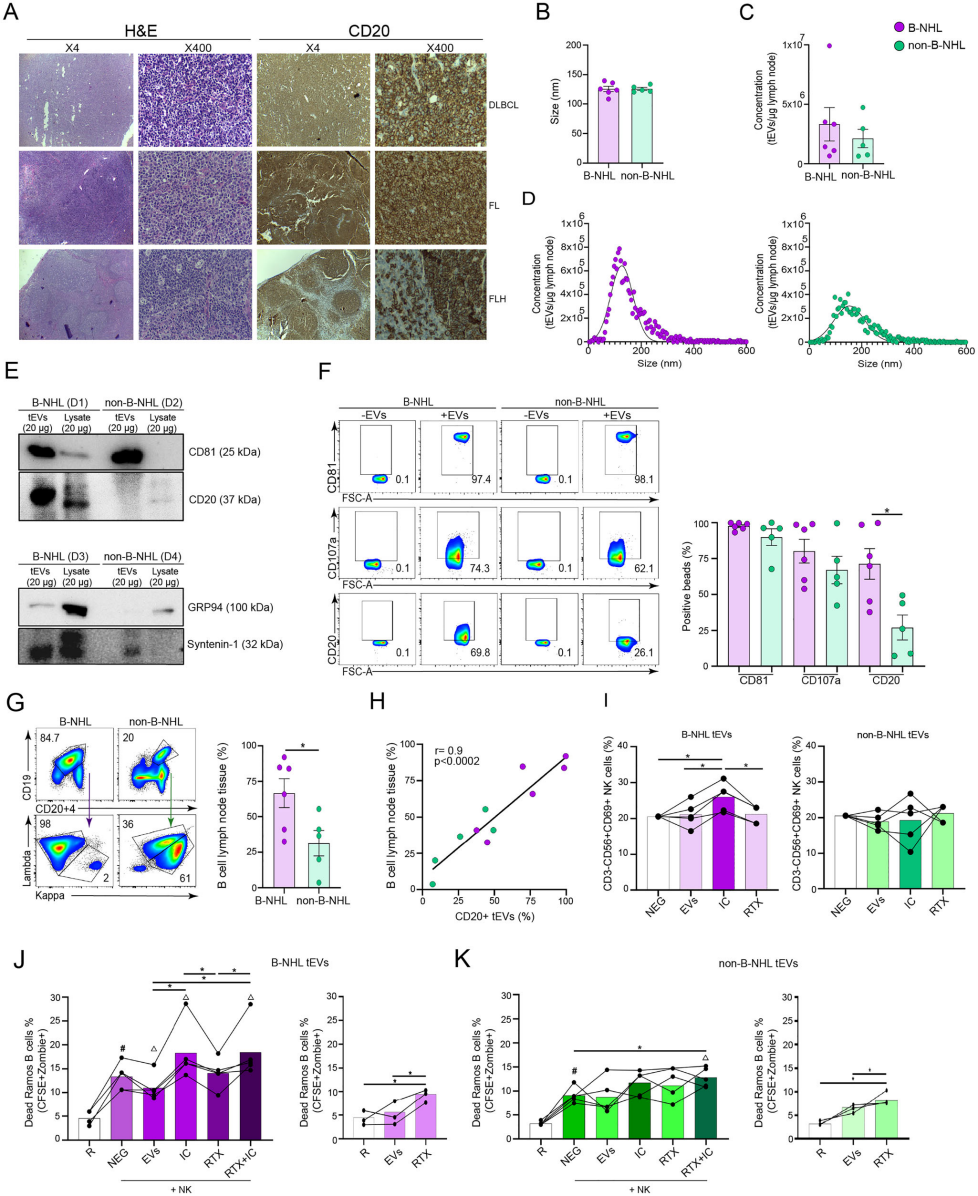
**CD20+ EVs from B‐NHL Lymph Node Explants Bind RTX and Activate NK Cells. (A)** Immunohistochemical analysis of representative lymphoid tissue samples. Left two columns display H&E staining illustrating the overall tissue architecture of DLBCL, FL, and FLH samples, respectively. Right two columns show CD20 immunostaining, highlighting B cell populations within the same samples. Images are shown at 4X and 400X. **(B‐D)** NTA characterization of EV size (nm, **B**), concentration (tEV/µg of tissue, **C**) and representative curve of size distribution (**D**) of B‐NHL (*n* = 6) and non‐B‐NHL (*n *= 5) tEVs. **(E)** Representative immunoblot characterization with antibodies directed against the EV markers CD81 and Syntenin‐1, non‐EV marker GRP94 and CD20 of tEVs from B‐NHL and non‐B‐NHL donors (20 µg). Cell lysates from lymph nodes (20 µg) were used as control. **(F)** Flow cytometry of tEVs from B‐NHL and non‐B‐NHL donors captured on anti‐CD63‐coated beads (CD81, CD107a and CD20). Representative dot plots (left) and bars showing the percentage of positive beads for each marker in tEVs from B‐NHL (*n *= 6) and non‐B‐NHL (*n *= 5) are shown. Latex beads without tEVs were used as negative control. **(G)** Flow cytometry analysis of lymph node explant cells showing the percentage of B cells and kappa:lamdba ratio in B‐NHL and non‐B‐NHL donors. Representative dot plots (left) and the percentage of B cells in B‐NHL (*n* = 6) and non‐B‐NHL (*n* = 5) donors are shown. **(H)** Correlation between the percentage of B cells in lymph node tissue and the percentage of CD20 in tEVs from paired samples depicted in figures F and G (*n *= 11). **(I)** Purified NK cells were stimulated with IL‐15 (5 ng/mL) and then exposed to ∼8.9 × 10^9^ tEVs (EVs) from B‐NHL (left) and non‐B‐NHL (right), ∼8.9 × 10^9^ tEVs + RTX (IC), RTX (1 µg/mL) or unexposed (NEG) for 4 h. The frequency of CD3‐CD56+CD69+ NK cells were analysed by flow cytometry (B‐NHL, *n *= 5 and non‐B‐NHL, *n *= 5). **(J–K)** Purified NK cells were exposed to ∼8.9 × 10^9^ tEVs (EVs) derived from either B‐NHL **(J)** or non‐B‐NHL **(K)** lymph nodes, ∼8.9 × 10^9^ tEVs pre‐incubated with RTX (IC), RTX (1 µg/mL), the combination of IC and free‐RTX (RTX+IC) or unexposed (NEG) for 30 min at 37°C. Thereafter, CFSE‐labelled Ramos cells were added at 5:1 NK: Ramos ratio. After 4 h, cells were labelled with Zombie NIR and analysed by flow cytometry. Percentage of cytotoxicity was calculated as 100 × percentage of CFSE+Zombie NIR high cells / percentage of CFSE+ cells. Frequency of CFSE+Zombie+ Ramos cells in the co‐cultures (*n* = 5, left) and the frequency of CFSE+Zombie+ Ramos cells alone and exposed to EVs or RTX (right) were used as controls. Spearman's correlation test was done in **H**. Mean ± SEM of n donors are shown in **B, C, F,** and **G**. *p* values were determined by Mann‐Whitney U test, Friedman and Wilcoxon matched‐pairs signed rank test. *p < 0.05. # p < 0.05 regarding Ramos cells without NK (R). Δ p < 0.05 regarding unmodulated cytotoxicity (NEG). Replicates in Figures **B‐H** correspond to different tissue donors; replicates in Figures **I‐K** correspond to different tissue donors and NK donors. B‐NHL (purple circle), non‐B‐NHL (green circle).

To validate our in vitro observations in patient‐derived samples, we analysed EVs released ex vivo from lymph node biopsies (tEVs) obtained from 21 donors. The cohort included 10 patients with untreated B‐NHL and 11 individuals with non‐malignant follicular lymphoid pathologies. Diagnostic classification was confirmed by histopathology and flow cytometric immunophenotyping (Table 1). Figure 3A shows representative haematoxylin‐eosin (H&E) and CD20 staining of tissue samples from DLBCL, FL and follicular lymphoid hyperplasia (FLH) patients.

Lymph node tissue explants were cultured for 24 h and tEVs were isolated from the culture supernatant by sequential ultracentrifugation. Despite some inter‐sample variability, a substantial fraction of cells remained viable after 24 h of culture (59.5 ± 7.6% viable cells, *n* = 7; data not shown), supporting the use of this ex vivo system for the analysis of tissue‐derived EVs. NTA revealed that the mean ± SEM diameter of tEVs was 125 ± 4.6 nm for B‐NHL samples and 126 ± 2.4 nm for non‐B‐NHL samples (Figure 3B). The concentration of particles released per µg of tissue was comparable between groups (3.3 ± 1.4 × 10^6^ for B‐NHL vs. 2.2 ± 0.7 × 10^6^ for non‐B‐NHL, Figure 3C) as well as the particle size distribution (Figure 3D). Immunoblot analysis showed that tEVs presented the EV markers CD81 and Syntenin‐1, while GRP94 was not detectable in tEVs from any of the two groups. As expected, CD20 levels were markedly higher in both tEVs and cell lysates from B‐NHL samples than in non‐B‐NHL controls, as demonstrated by immunoblot (Figure 3E) and bead‐based flow cytometry (Figure 3F). Importantly, the EV markers CD81 and CD107a showed similar staining levels in both groups of donors. These results suggest that B cell lymphoma secrete EVs with high levels of CD20 on their surface, making them potential targets for RTX binding and immune complex formation.

To further investigate whether EVs reflect the cellular composition of lymphoma tissues, we quantified the frequency of CD20+ B cells in lymph node biopsies and compared it with the abundance of CD20+ EVs released into the corresponding explant culture supernatants. Representative flow cytometry plots illustrating the percentage of CD19+CD20+ B cells in a B‐NHL sample and a non‐B‐NHL control are shown in Figure 3G (left). Kappa and lambda light chain expression by B cells showing clonality is also illustrated. We found that B‐NHL patients had a significantly higher percentage of total B cells in tissue (66.7 ± 10.0%, including atypical and normal B cells) compared to non‐B‐NHL patients (31.4 ± 8.9%, *p* < 0.05; Figure 3G, right). Remarkably, the percentage of CD20+ tEV‐positive beads strongly correlated with the frequency of B cells in the corresponding lymph node tissue (*r* = 0.9, *p* < 0.0002; Figure 3H). These findings indicate that the abundance of CD20+ EVs released ex vivo reflects the burden of CD20+ cells within B‐NHL tissues, supporting the notion that tissue‐derived EVs preserve key phenotypic characteristics of their cells of origin.

To determine whether our previous observations extended to patient‐derived samples, we evaluated whether tissue‐derived tEVs released *ex vivo* from lymph node explants recapitulated the RTX‐dependent modulation of NK cell activity observed with Ramos‐derived EVs. We observed that the addition of tEVs derived from B‐NHL to NK cells did not modify NK cell activation, as revealed by CD69 surface exposure determined by flow cytometry analysis (Figure 3I). Notably, the formation of tEV‐RTX immune complexes led to a significant increase in NK cell CD69 surface expression, whereas RTX alone failed to induce NK cell activation (*p* < 0.05; Figure 3I, left). Interestingly, when tEVs derived from non‐B‐NHL were added to NK cells, the percentage of CD56+ cells expressing CD69 remained stable among different conditions (Figure 3I, right).

Finally, we evaluated whether B‐NHL‐derived tEV‐RTX immune complexes could modulate the cytotoxic activity of purified NK cells using Ramos cells as targets. Consistent with our observations using Ramos‐derived EVs, exposure to B‐NHL tEVs significantly reduced NK cell cytotoxicity (10.9 ± 1.2%) compared with baseline levels (13.4 ± 1.3%, *p* < 0.05). In contrast, tEV‐RTX immune complexes significantly enhanced NK cell cytotoxicity (18.3 ± 2.6%, *p* < 0.05), fully reversing the inhibitory effect exerted by tEVs (Figure 3J). Notably, the addition of soluble RTX to tEV‐RTX immune complexes did not further increase NK cell cytotoxicity, indicating that the stimulatory effect was primarily mediated by the immune complexes themselves. In contrast, non‐B‐NHL tEVs did not modulate NK‐mediated cytotoxicity either alone or pre‐incubated with RTX (Figure 3K), and only with the combined RTX and tEV‐RTX treatment a significant but modest enhancement was observed.

These findings indicate that only EVs derived from B‐NHL, when specifically recognized by RTX, can enhance NK cell activation. This effect is likely driven by the elevated abundance of CD20+ EVs released by malignant B cells, which enables efficient formation of RTX‐EV immune complexes capable of engaging and activating NK cells.

### 3.4 Elevated Plasma CD20+ EVs in B‐NHL Patients Modulate NK Cell Activation

We hypothesized that EVs produced by malignant B cells in lymph nodes enter the circulation, allowing tumour‐associated changes in EVs production to be reflected in the blood. This provides a rationale to study the formation and functional impact of immune complexes generated in the bloodstream following RTX infusion.

**FIGURE 4:**
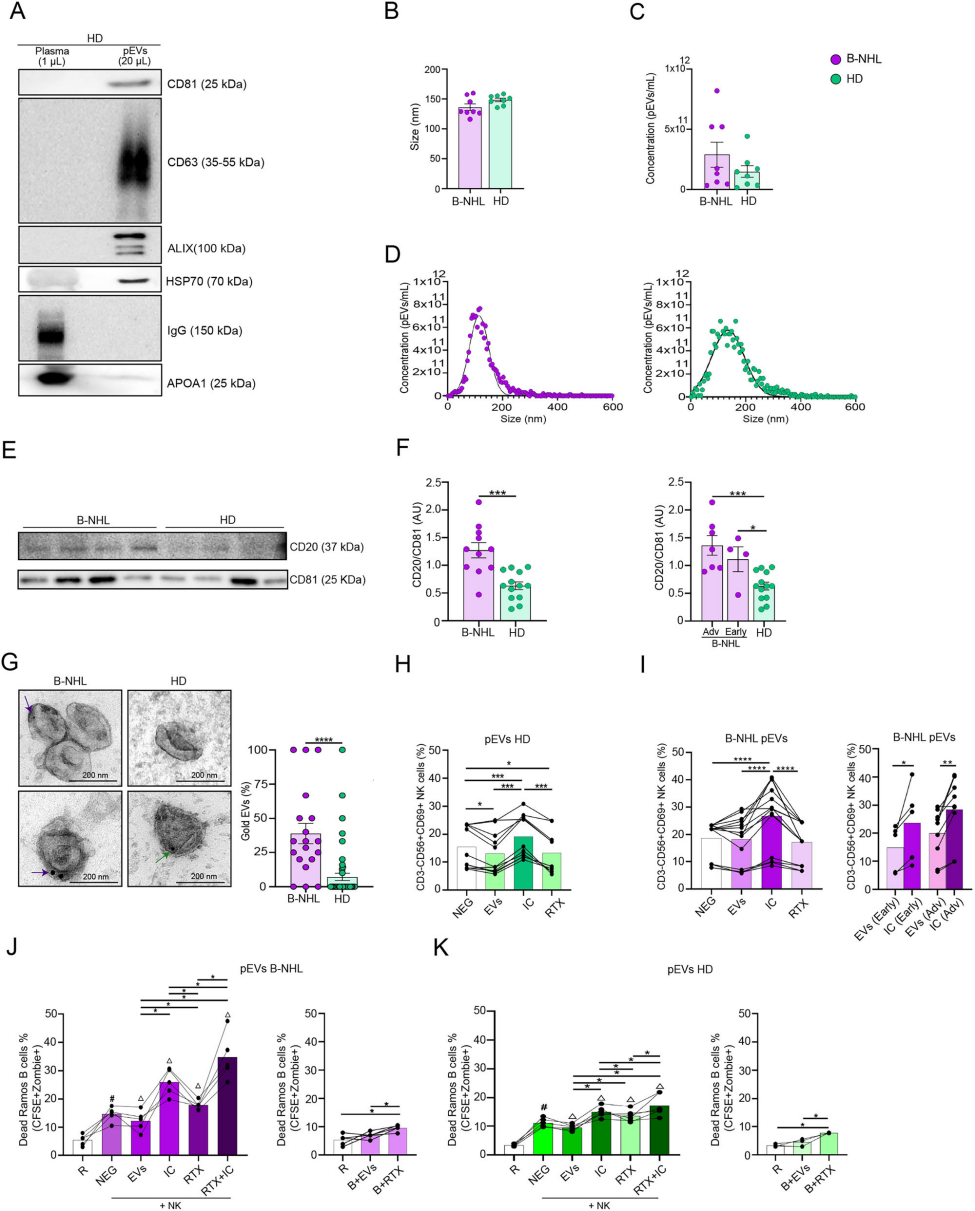
**CD20+ pEVs from B‐NHL patients bind RTX creating immune complexes which modulate NK cell activation**. **(A)** Representative immunoblot characterization of pEVs (20 µL) from a healthy donor (HD) using antibodies directed against the EV markers CD81, CD63, ALIX and HSP70, and non‐EV marker IgG and APOA1. Whole plasma was used as control (1 µL). **(B‐D)** NTA characterization of pEV size (nm, **B**), concentration (pEV/mL of plasma, **C**) and representative curve of size distribution **(D)** of B‐NHL (*n* = 8) and HD (*n* = 8). **(E)** Representative immunoblot characterization of pEVs from B‐NHL (*n* = 4) and HD (*n* = 4) showing CD81 and CD20 levels. **(F)** Densitometric analysis showing CD20/CD81 ratio in pEVs from B‐NHL (*n* = 11) and HD (*n* = 13; left). CD20/CD81 ratio in pEVs from B‐NHL divided according to their disease stage (advanced *n* = 7, early *n* = 4) and HD (*n* = 13; right). **(G)** TEM analysis of pEVs from B‐NHL and a HD showing protein A‐immunogold staining to detect the binding of RTX on the membrane of pEVs (scale 200 nm; *n *= 2 from each group). Representative pictures (left) and percentage of gold particles associated with EVs observed into two grids over more than 100 EVs (right) are shown. **(H‐I)** Purified NK cells were stimulated with IL‐15 (5 ng/mL) and then exposed to ∼1.1 × 10^9^ pEVs (EVs) from HD and/or B‐NHL, ∼1.1 × 10^9^ pEVs + RTX (IC), RTX (1 µg/mL) or unexposed (NEG) for 4 h. The frequency of CD3‐CD56+CD69+ NK cells was analysed by flow cytometry. Frequency of CD3‐CD56+CD69+ cells in HD (*n *= 11, **H**) B‐NHL (*n *= 14, **I** left) and B‐NHL stratified by early (*n *= 5) or advanced (*n *= 9) stage of disease (**I** right) are shown. **(J, K)** Purified NK cells were exposed to ∼1.1 × 10^9^ plasma EVs derived from B‐NHL patients **(J)** or HD **(K)**, ∼1.1 × 10^9^ pEVs pre‐incubated with RTX (IC), RTX (1 µg/mL), the combination of IC and free‐RTX (RTX+IC) or unexposed (NEG) for 30 min at 37°C. Thereafter, CFSE‐labelled Ramos cells were added at 5:1 NK: Ramos ratio. After 4 h, cells were labelled with Zombie NIR and analysed by flow cytometry. Percentage of cytotoxicity was calculated as 100 × percentage of CFSE+Zombie NIR high cells / percentage of CFSE+ cells. Frequency of CFSE+Zombie+ Ramos cells in the co‐cultures (*n *= 5; left). Ramos cells alone and exposed to EVs or RTX (right) were used as controls. Mean ± SEM of donors are shown in **B**, **C, F**, and **G**. *p* values were determined by Mann‐Whitney *U* test, Kruskal–Wallis test, Friedman and Wilcoxon matched‐pairs signed rank test. *p < 0.05, **p < 0.01, ***p < 0.001 and ****p < 0.0001. #*p* < 0.05 regarding Ramos cells without NK (B). Δ *p* < 0.05 regarding unmodulated cytotoxicity (NEG). Replicates in Figures **B‐F** correspond to different plasma donors; replicates in Figures **I‐K** correspond to different plasma donors and NK donors. B‐NHL (purple circle), HD (green circle).

EVs from B‐NHL and HD were isolated using SEC, as previously described (Adamczyk et al. 2023). Characterization on 1 mL size exclusion chromatography (SEC) elution fractions showed enrichment of EVs in fractions 5–6 (Figure S3A‐B). Accordingly, these fractions were combined and further concentrated by centrifugation to obtain plasma EVs (pEVs) preparations. Immunoblot analysis performed using plasma samples obtained from HD confirmed that pEV preparations were enriched in the classical EV markers CD63 and CD81, as well as ALIX and HSP70, compared to plasma (Figure 4A). In contrast, IgG and APOA‐1 were substantially less abundant in the pEV fractions. These findings indicate that our pEV preparations were not significantly contaminated with plasma components, confirming their purity. NTA revealed that the mean ± SEM diameter of pEVs was 136.0 ± 5.6 nm in B‐NHL and 148.0 ± 2.8 nm in HD (Figure 4B). The concentration of particles per mL of plasma (2.8 ± 1.1 × 10^11^ for B‐NHL vs. 1.7 ± 0.5 × 10^11^ for HD) and the size distribution was comparable between groups (Figure 4C‐D).

Then, we analysed the abundance of CD20+ pEVs in B‐NHL. Representative immunoblots are shown in Figure 4E. Interestingly, B‐NHL patients had a higher abundance of CD20+ pEVs than HD, as evidenced by a significant increase in the CD20/CD81 index detected by western blot (*p* < 0.0001, Figure 4F, left panel). Moreover, patients in more advanced stages of B‐NHL exhibited the highest CD20/CD81 index (*p* < 0.0001 and *p* < 0.05 for advanced and early stages vs. HD, respectively; Figure 4F, right panel).

Transmission electron microscopy with immunogold labelling was performed on pEVs from B‐NHL patients and HD. All samples were previously incubated with RTX. Representative image of pEVs from a B‐NHL and a HD are shown (Figure 4G, left panel). A higher frequency of gold‐labelled EVs was observed in the B‐NHL samples (38.9 ± 7.3%) compared to HD (7.0 ± 2.6%, mean ± SEM; *p* < 0.0001), indicating that the increased expression of CD20 may facilitate greater binding of RTX to these EVs (Figure 4G, right panel).

Next, we sought to determine whether the RTX‐dependent modulation of NK cell activity observed with Ramos and tissue‐derived EVs could also be detected in circulating plasma EVs. We observed that the addition of pEVs derived from HD to NK cells resulted in a reduction of the percentage of CD56+ cells expressing CD69 (13.0 ± 2.2%) compared to unexposed (NEG) NK cells (15.4 ± 2.0%, *p* < 0.05; Figure 4H). Notably, this decrease in activation was reversed in the presence of the EVs‐RTX immune complex, with CD69+ expression increasing to 19.2 ± 2.3% (*p* < 0.001). In contrast, exposure to RTX alone did not significantly enhance CD69 expression, which remained low at 13.2 ± 2.2%, significantly lower than the effect observed with the immune complex (*p* < 0.001). Interestingly, when pEVs derived from B‐NHL were added to NK cells, there was no reduction in the percentage of CD56+ cells expressing CD69 (18.6 ± 2.0%) compared to unexposed NK cells (18.1 ± 2.3%; Figure 4I, left panel). However, the presence of the EVs‐RTX immune complex markedly increased CD69+ expression to 26.7 ± 3.2% (*p* < 0.0001). Conversely, RTX alone did not significantly alter CD69 expression, which remained similar to the control (17.1 ± 1.8%) and was significantly lower than the effect induced by the immune complex (*p* < 0.0001). Similar trends were observed when patients were stratified according to disease stage (Figure 4I, right panel).

Finally, we assessed the impact of pEV‐RTX immune complexes on NK‐cytotoxic activity using Ramos as target cell line. In line with previous results, pEVs derived from B‐NHL patients diminished NK cell cytotoxicity (12.2 ± 1.6%) compared to baseline (14.6 ± 1.1%, *p* < 0.05), while B‐NHL pEV‐RTX immune complexes significantly augmented NK‐cytotoxic activity (26.0 ± 2.1%, *p* < 0.05) (Figure 4J). Interestingly, combination of RTX and pEV‐RTX immune complexes again resulted in an additive effect on NK‐cytotoxicity (34.0 ± 3.8%, *p* < 0.05; Figure 4J), as previously observed for Ramos‐derived EVs (Figure 2D). Regarding pEVs from healthy donors, they induced a decreased NK cell cytotoxic activity (9.5 ± 0.4%, *p* < 0.05) compared to baseline (11.1 ± 0.6%, *p* < 0.05; Figure 4K). In accordance with their positive CD20 expression, HD pEV‐RTX immune complexes increased NK‐cytotoxicity (14.9 ± 0.8%, *p* < 0.05) although to a lower magnitude than B‐NHL pEVs. These findings are consistent with the notion that higher levels of CD20+ EVs are associated with stronger NK cell activation induced by EV‐RTX immune complexes, corroborating the results obtained with tissue‐derived EVs.

## 4 Discussion

Our study reveals that CD20+ EVs produced by B cell lymphomas serve as targets for rituximab. Importantly, RTX binding fundamentally alters the biological activity of these vesicles, converting them from suppressors of NK cell activation and cytotoxicity into immune‐stimulatory platforms capable of promoting FcγR‐dependent NK cell responses. These findings uncover a novel mechanism through which RTX may contribute to anti‐tumour immunity, namely, the formation of immune complexes with tumour‐derived EVs that enhance NK cell activation and cytotoxicity. More broadly, our results suggest that therapeutic antibodies can reshape the immunological properties of EVs, revealing an additional layer of interaction between antibody‐based therapies and the tumour microenvironment.

We provide compelling evidence that EVs released from B‐NHL cells (including the Ramos cell line, primary lymph node tissue, and patient plasma), not only express high levels of CD20 on their surface but also expose the specific epitope recognized by RTX. These findings suggest that, following administration, RTX can bind not only malignant B cells but also CD20+ EVs, extending its range of targets to the extracellular vesicle compartment. Importantly, the resulting EV‐RTX immune complexes are functionally active: their engagement of FcγRIIIa on NK cells triggers intracellular signalling cascades, including Syk phosphorylation, leading to enhanced NK cell activation and cytotoxicity.

This observation underscores a previously unrecognized mechanism by which RTX transforms CD20+ tumour‐derived EVs from immunosuppressive or inert entities into immune‐stimulatory structures. These findings align with prior reports showing that plasma‐derived EVs from both treatment‐responsive and refractory/relapsed DLBCL patients impair NK cell function (Zare et al. 2020). Although those studies characterized the immunosuppressive effects of lymphoma‐derived EVs, they did not address how therapeutic antibody binding influences EV activity. Our data suggest that, beyond its established mechanisms of action, including CDC, ADCC, and direct induction of tumour cell death, RTX can also reshape the immunological properties of circulating and tissue‐derived EVs, thereby enhancing NK cell‐mediated immune surveillance.

Although the enhancement of NK‐cell activity induced by EV–RTX immune complexes was consistently observed across all donors tested, the magnitude of the response varied between individuals. One factor that may contribute to this variability is genetic heterogeneity in FcγRIIIa. Previous studies have shown that the FCGR3A‐158 V/F polymorphism modulates the affinity of FcγRIIIa for IgG1 antibodies, with carriers of the 158 V allele generally exhibiting stronger ADCC responses and improved clinical outcomes following antibody‐based therapies than individuals homozygous for the 158F allele (Koene et al. 1997; Cartron et al. 2002; Cuello et al. 2016). Although FCGR3A genotyping was not available for the donors included in this study, precluding formal genotype‐response analyses, this polymorphism represents a plausible contributor to the inter‐individual differences observed in NK cell responses. Future studies incorporating prospective FCGR3A genotyping will be important to determine whether the immunostimulatory activity of EV–RTX immune complexes is influenced by FcγRIIIa genetic variants and whether this mechanism contributes to the heterogeneous clinical responses observed in RTX‐treated patients.

Previous reports proposed that CD20+ EVs contribute to resistance against anti‐CD20 therapy by acting as antibody decoys, sequestering RTX and limiting its availability for malignant B cells (Aung et al. 2011). Our study adds a new layer of complexity by demonstrating that the sequestration of RTX by EVs leads to the formation of stimulatory immune complexes that enhance NK cell activity.

Several studies have documented increased CD20 expression in tumour cells from individuals with B‐NHL compared to non‐malignant B cell populations (Horna et al. 2019; Johnson et al. 2009; Prevodnik et al. 2011), and lower CD20 expression has been associated with poorer response to RTX therapy (Prevodnik et al. 2011). In agreement with these findings, we show that plasma from B‐NHL patients contains elevated levels of CD20+ EVs compared to healthy donors, particularly in advanced‐stage disease. A key strength of our study is the inclusion of lymph node explants from B‐NHL patients, which enabled the direct analysis of EVs released by primary lymphoma tissues ex vivo. These tissue‐derived EVs were enriched in canonical EV markers and CD20, providing a clinically relevant system that bridges observations made in cell lines and circulating plasma EVs. Notably, the abundance of CD20+ EVs correlates strongly with B cell infiltration in these tissues, validating lymphoid tissue as a relevant and active source of EVs. This observation has two potentially important implications. First, the amount of EVs produced by the tumour and released into the circulation may influence whether the “sequestering” of RTX or the immunostimulatory effects of EV‐RTX immune complexes predominate. Second, it suggests that EV profiling could serve as a surrogate for characterizing the immunophenotypic features of the tumour microenvironment, as it has been previously shown in other settings (Hoshino et al. 2020). This could be particularly relevant to determine response to therapy. However, it is important to note that CD20 is also expressed by normal mature and memory B cells in both lymphoid tissues and circulation. Consequently, CD20+ EVs are not exclusively tumour‐derived, limiting their specificity as lymphoma biomarkers. In line with this notion, we observed that RTX immune complexes formed with EVs from healthy donors were also capable of enhancing NK cell cytotoxicity, indicating that the biological effect described here is not restricted to malignant EVs. Rather, our findings suggest that the formation of EV‐RTX immune complexes represents a general immunological mechanism that can occur whenever CD20+ EVs are present. Importantly, B‐NHL patients exhibited substantially higher levels of CD20+ EVs than healthy individuals, and this increase was accompanied by a stronger enhancement of NK cell activity following RTX binding. These observations indicate that the magnitude of the response is directly related to the abundance of CD20+ EVs available for immune complex formation.

The implications of our findings may extend beyond B cell lymphoma. Because EVs frequently display antigens derived from their cells of origin, they can interact with cognate antibodies and influence immune responses. Similar EV‐antibody interactions have been reported in infectious diseases, autoimmunity, and cancer (Troyer et al., 2021; Nielsen et al. 2012, 2015; Edo et al., 2019; Simon et al., 2018). Our study demonstrates that such interactions are not merely passive binding events but can profoundly alter EV biological activity, generating immune complexes that enhance NK cell responses.

Despite the novel insights provided by our study, several limitations should be acknowledged. First, although we demonstrated that CD20+ EVs form immune complexes with RTX and enhance NK cell activation in vitro, the in vivo functional consequences of these complexes remain to be validated in clinical settings or relevant animal models. Second, while we showed that CD20+ EV levels correlate with tissue B cell infiltration and increase in the plasma of B‐NHL patients, the specificity of CD20 as a biomarker is limited due to its expression on normal B cells. Additionally, the heterogeneity among B‐NHL subtypes and disease stages might influence EV composition and immunological behaviour, and larger patient cohorts are needed to fully assess the clinical relevance of CD20+ EVs and their interaction with therapeutic antibodies.

In summary, our findings reveal that CD20+ EVs derived from B‐NHL can bind RTX, forming immune complexes that activate NK cells via FcγRIIIa. This uncovers a previously unrecognized immunostimulatory mechanism of RTX, extending its action beyond direct tumour targeting. Importantly, our data also demonstrate that EVs can undergo a functional switch upon antibody recognition: whereas EVs produced by tumour cells tend to suppress NK cell activity, EVs specifically recognized by RTX acquire immunostimulatory properties, highlighting a dual role of EVs in both immune evasion and immune activation. This unveils new opportunities for strategies based on antibody‐vesicle recognition as candidates for immunomodulatory therapies.

## Author Contributions

**Adrián V. Otero**: investigation, methodology, validation, visualization, writing – review and editing, formal analysis, data curation. **Paula Soledad Pérez**: methodology, validation, visualization, writing – review and editing, data curation, supervision. **Belen Gaete‐ramírez**: investigation. **Gregorio Cordini**: resources. **Matías Norte**: resources. **Enriqueta Martínez**: resources. **Nicolás Marsol**: resources. **Cecilia Malusardi**: resources, formal analysis, investigation. **Sofía Rivarola**: resources. **Ana Cantillo**: resources. **María Luz Leicaj**: methodology, validation. **Constanza Russo**: methodology, validation. **Juan Quarroz Braghini**: investigation, formal analysis, visualization. **Federico Penas**: methodology, validation. **Glenda Ernst**: project administration. **Cecilia Cabral**: investigation, visualization, formal analysis. **Silvina Palmer**: resources, conceptualization. **Manuel Varas‐Godoy**: writing – review and editing, resources, supervision, funding acquisition, conceptualization. **Lourdes Arruvito**: conceptualization, writing – original draft, funding acquisition, writing – review and editing, project administration, supervision, data curation, formal analysis. **Matías Ostrowski**: conceptualization, funding acquisition, writing – original draft, writing – review and editing, project administration, supervision.

## Funding

This work was supported by the National Agency of Research and Development (ANID): “Financiamiento Basal para Centros Científicos y Tecnológicos de Excelencia Centro Ciencia & Vida” Basal FB210008 and Fondecyt 1230983 (Manuel Varas‐Godoy) and by the Argentinean National Agency for the Promotion of Science and Technology grants PICT 2020‐0261 (Lourdes Arruvito) and PICT 2019–02506 (Matias Ostrowski).

## Conflicts of Interest

The authors have declared that no conflict of interest exists.

## Supporting information




**Supplementary Figure 1. Comparison of pEV recovery after centrifugation at 30,000 g and 100,000 g**. Immunoblot showing Syntenin‐1, CD9 and IgG in centrifuged EVs.

**Supplementary Figure 2. Trastuzumab binding to cell lines**. Histograms showing trastuzumab binding on the HER2+ breast cancer cell line BT‐474 (left) revealed by secondary staining with anti‐human‐Fc IgG. As expected, Ramos cells were not stained by trastuzumab (right).

**Supplementary Figure 3. Isolation of pEVs by SEC. (A)** EVs were isolated from plasma samples by SEC and the first 9 elution fractions (1 mL each) were concentrated at 30,000 *g* and pellets were analyzed by immunoblot for the EV markers Syntenin‐1 and CD9 and the plasma protein IgG. **(B)** Quantification of total protein by BCA (left) and particle count by NTA (right) in the first 9 elution fractions from the SEC column.

## Data Availability

The data that support the findings of this study are available from the corresponding author upon reasonable request.
